# Decreased expression of pseudogene *PTENP1* promotes malignant behaviours and is associated with the poor survival of patients with HNSCC

**DOI:** 10.1038/srep41179

**Published:** 2017-01-23

**Authors:** Jiannan Liu, Yue Xing, Liqun Xu, Wantao Chen, Wei Cao, Chenping Zhang

**Affiliations:** 1 Department of Oral Maxillofacial-Head and Neck Oncology, Ninth People’s Hospital, Shanghai Jiao Tong University School of Medicine, Shanghai 200011, P.R. China; 2 Shanghai Research Institute of Stomatology and Shanghai Key Laboratory of Stomatology, Shanghai 200011, China; 3 Department of Ophthalmology, Ninth People’s Hospital, Shanghai Jiao Tong University School of Medicine, Shanghai 200011, P.R. China

## Abstract

*PTENP1*, a pseudogene of *PTEN*, was previously reported to be a tumour suppressor in some cancer types. However, there was no evidence for the biological function and expression of *PTENP1* in head and neck squamous cell carcinoma (HNSCC). Here, we evaluated the function and clinical implications of *PTENP1* in HNSCC. Using RT-PCR and quantitative real-time PCR (qRT-PCR), we found that the level of *PTENP1* was reduced in HNSCC specimens compared with adjacent tissues. A decrease in the *PTENP1* copy number, but not in the PTEN copy number, was frequently observed in tumour cell lines (4 of 5 cell lines) by genomic real-time PCR. Decreased *PTENP1* expression was significantly associated with a history of alcohol use (P = 0.034). Univariate and multivariate Cox regression analyses revealed that low expression of *PTENP1* correlated with worse overall survival (OS, P = 0.005; HR:0.170; Cl:0.049 to 0.590) and disease-free survival (DFS, P = 0.009; HR:0.195; Cl:0.057 to 0.664) rates of HNSCC patients. Furthermore, ectopic *PTENP1* expression inhibited the proliferation, colony formation and migration of HNSCC cells and the growth of xenograft HNSCC tumours. These results demonstrate that *PTENP1* might play an important role in the initiation and progression of HNSCC.

Head and neck squamous cell carcinoma (HNSCC) is the most common malignant lesion in the head and neck region, with an approximate incidence rate of 135.1 per 100,000 people in China each year^1^. HNSCC is a heterogeneous tumour with an aggressive phenotype and a poor prognosis due to local recurrence and locoregional lymph node metastasis^2^. A better understanding of the genetic and epigenetic molecular alterations of the disease is critical to providing the appropriate treatment and improving the survival of patients with HNSCC.

LncRNAs are transcripts of more than 200 nucleotides without protein-coding function^3^. The non-coding RNAs, which are not transcribed from protein-coding genes, constitute a large portion of the mammalian transcriptome^4^,^5^. LncRNAs exist in many types of transcripts, including antisense RNA, small nucleolar RNA, enhancer RNA, endogenous RNA, intergenic transcripts and RNA overlapping the exon transcripts^6^,^7^,^8^,^9^. Up to now, lncRNA has been described as a key address code, orchestrating the trafficking of protein complexes, genes, chromosomes and also RNAs to appropriate locations and ensuring that they are subject to proper activation or suppression^9^,^10^. Recently, lncRNAs were reported to be involved in various biological processes and to exert great influence on disease development, especially cancers. The well-known lncRNA, metastasis-associated lung adenocarcinoma transcript 1 (*MALAT-1*), is highly expressed in early-stage non-small-cell lung carcinoma and could predict the metastasis and prognosis of these patients^11^. Several lncRNAs were also found to be promising prognostic markers for other cancer types such as melanoma, prostate cancer, and kidney cancer^12^,^13^,^14^. Recently, a landscape of lncRNAs expression has been identified to reveal the lncRNAs that are significantly differentially expressed between the oral mucosa and oral premalignant lesions^15^. Although a large number of human lncRNAs have been identified, the biological functions of these lncRNAs remain largely unknown, especially in cancers.

LncRNA *PTENP1* is the pseudogene of the PTEN, which is a tumour suppressor gene (TSG). It is highly homologous to PTEN, sharing 98% sequence identity with the the PTEN mRNA sequence^16^,^17^. An increasing number of studies have shown that *PTENP1* functions as a competing endogenous RNA to suppress tumour progression^18^. However, the expression and biological function of *PTENP1* in HNSCC have not yet been elucidated. Here, we evaluated the possible function of *PTENP1* and found that it acts as a potential tumour suppressor due to a reduction in the copy number, independent of PTEN, in HNSCCs and can serve as an independent prognostic factor in patients with HNSCC.

## Results

### The expression pattern and cellular sublocalization of lncRNA *PTENP1* in HNSCC specimens and HNSCC cell lines

**Figure 1:**
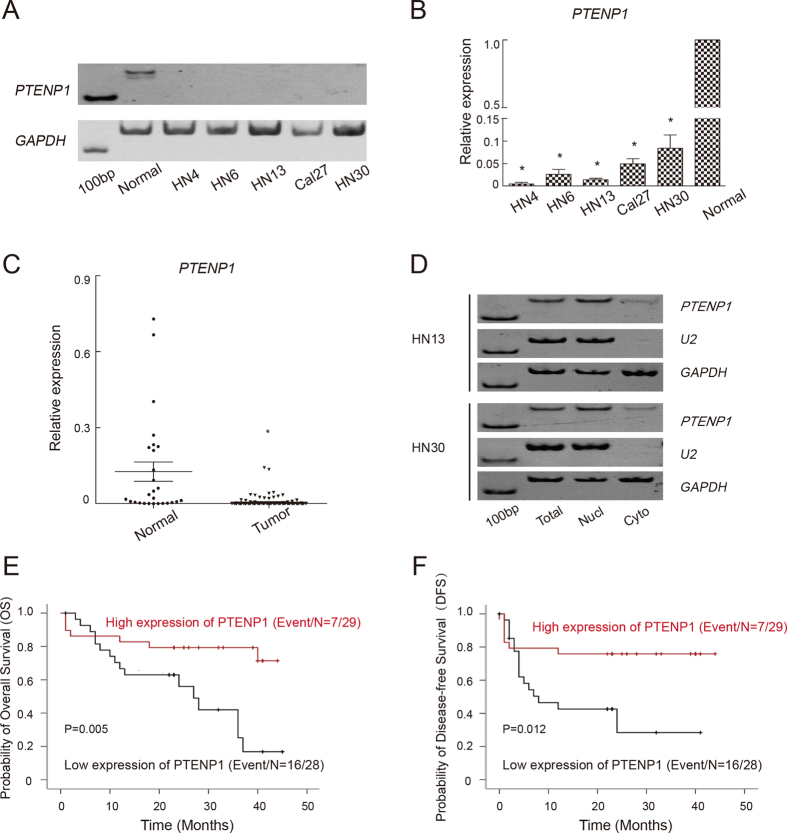
The expression pattern and clinical implications of *PTENP1* in HNSCCs. (**A**). RT-PCR was performed to determine the expression of *PTENP1* in five HNSCC cell lines. Oral mucosal epithelial cells were used as a control. The full-length gels are presented in Supplementary Figure 1A and B, (**B**). The results of a real-time PCR analysis of the *PTENP1* expression in five HNSCC cell lines. The expression level in oral mucosal epithelial cells was normalized to 1. The data were presented as the means ± SD. *p < 0.05. (**C**) The results of a real-time PCR analysis of the *PTENP1* expression in normal and tumour tissue specimens. *p < 0.05. (**D**) The cellular location of *PTENP1* in HN13 and HN30 cells. *U2* was used as a positive control for nuclear RNA. *GAPDH* was used as a positive control for cytoplasmic RNA. The full-length gels are presented in Supplementary Figure 1C–E. (**E**,**F**) Kaplan-Meier survival curve indicated overall survival (OS, (**E**)) and disease-free survival (DFS, (**F**)) by evaluation of the expression levels of *PTENP1* in the cohort.

We first examined the expression of lncRNA *PTENP1* in five HNSCC cell lines by RT-PCR and real-time PCR (Fig. 1A and B). Compared with normal oral mucosal epithelial cells, *PTENP1* expression was reduced in all five HNSCC cell lines. We then detected the expression level of *PTENP1* in 57 HNSCC tissues and 27 adjacent normal tissues (Fig. 1C). Consistent with the results in cell lines, *PTENP1* expression was decreased in HNSCCs compared to adjacent normal tissues (*P* < 0.01).

To address the cellular sublocalization of *PTENP1*, we next examined the distribution of *PTENP1* in two randomly selected HNSCC cell lines using two positive control genes: *U2* small nuclear RNA, which is mainly found in nuclei and *GAPDH* mRNA, which is mainly present in the cytoplasm. In both HN13 and HN30 cells, *PTENP1* mainly existed in the nucleus (Fig. 1D).

### Decreased *PTENP1* expression was associated with a history of alcohol use and a worse clinical outcome

Our findings showed that low *PTENP1* transcript levels were significantly correlated with a history of alcohol use (*P* = 0.034) (Table 1) and a worse OS (*P* = 0.005) or DFS (*P* = 0.012) (Fig. 1E and F). However, there were no significant associations between the *PTENP1* level and age, gender, smoking history, disease site, tumour status, tumour size, TNM stage, tumour stage or lymph node metastasis of HNSCCs patients (Table 1). The univariate COX proportional Hazards regression analysis showed that the *PTENP1* level was an independent predictor of the OS (*P* = 0.005; HR:0.170; Cl:0.049 to 0.590) and DFS (*P* = 0.009; HR:0.195; Cl:0.057 to 0.664) in patients with HNSCC (Table 2).

### Positive correlation between *PTENP1* and PTEN in HNSCCs

**Figure 2:**
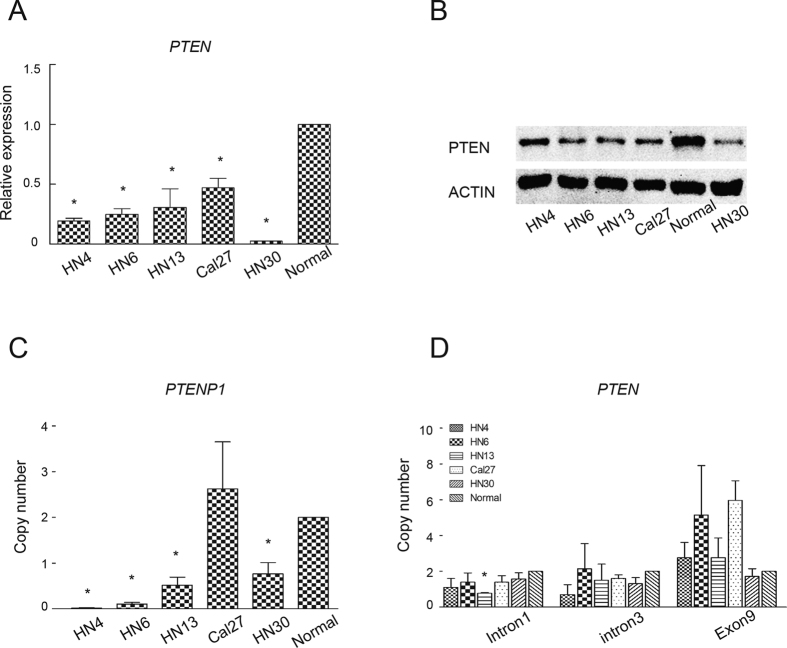
Expression of PTEN and copy number of *PTENP1* or PTEN were detected in HNSCC cells. (**A**) The results of a real-time PCR analysis of the PTEN expression in five HNSCC cell lines. The expression of oral mucosal epithelial cells was normalized to 1. The data were presented as the means ± SD. *p < 0.05. (**B**) Western blot of PTEN expression in the five HNSCCs cell lines. Oral mucosal epithelial cells were used as a control. The full-length blots are presented in Supplementary Figure 1F and G. (**C**) Genomic real-time PCR of the *PTENP1* copy number in five HNSCC cells. The data were presented as the means ± SD. *p < 0.05. (**D**) Genomic real-time PCR of the PTEN copy number in five HNSCC cells. Primers were designed against intron 1, intron 3 and exon 9 of PTEN. The data were presented as the means ± SD. *p < 0.05.

Because PTEN was reported to be the target gene protected by *PTENP1*, we also detected the expression of PTEN in tumour cells. We found that the PTEN expression was significantly decreased in all five HNSCCs cell lines as determined by real-time PCR (Fig. 2A). In a Western blot assay, PTEN showed lower expression in HNSCC cell lines compared with normal oral mucosal epithelial cells (Fig. 2B).

### Copy number reduction in lncRNA *PTENP1* from the genome in HNSCCs

Since genomic *PTENP1* and PTEN were previously found to be decreased in terms of the copy number in human melanoma, we also examined the genomic status of these two genes in HNSCC cell lines as reported in the previous study^19^. A genomic qPCR analysis of *PTENP1* revealed complete deletion in HN4 cells, partial deletion in the HN6, HN13 and HN30 cell lines, and no deletion in the Cal27 cell line (Fig. 2C). An analysis of PTEN intron 1, intron 3 and exon 9 revealed no significant deletion in the HN4, HN6, HN30 or Cal27 cell lines. However, there was a partial deletion of PTEN intron 1 in the HN13 cells (Fig. 2D). These results suggest that deletion of the genomic *PTENP1* is a frequent event in HNSCCs, but deletion of genomic PTEN is not common in HNSCCs.

### *PTENP1* was not sufficient to completely recover the level of PTEN in HNSCCs

**Figure 3:**
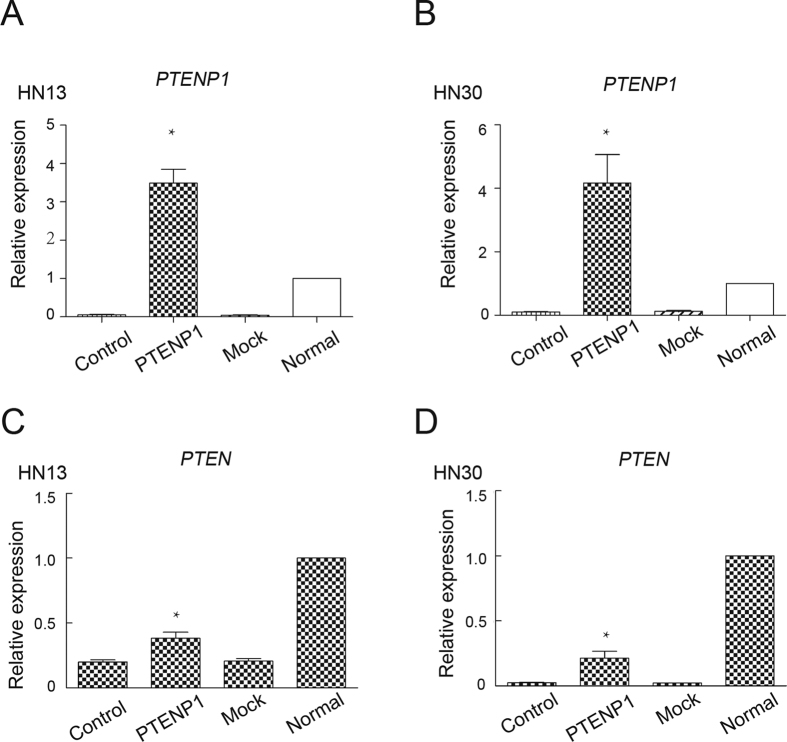
Co-expression of *PTENP1* and PTEN in HNSCC cells. (**A**,**B**) The results of a real-time PCR analysis of the *PTENP1* expression after PCMV-*PTENP1* transfection in HN13 and HN30 cells. The data were presented as the means ± SD. *p < 0.05. (**C**,**D**) The results of a real-time PCR analysis of the PTEN expression after PCMV-*PTENP1* transfection in HN13 and HN30 cells. The data were presented as the means ± SD. *p < 0.05.

There is high homology between PTEN and *PTENP1*^20^. To better understand the potential functions of *PTENP1*, we established an over-expression plasmid for *PTENP1*. The plasmid was packed into a lentivirus, which was then transfected into the HN13 and HN30 cell lines. Cells carrying the empty vector plasmids were used as mock controls. We examined the expression of *PTENP1* in stable cell lines by real-time PCR (Fig. 3A and B). Compared with the wild type control and PCMV mock control, the expression of *PTENP1* was notably increased in HNSCCs. We also examined the expression of PTEN in these stable cell lines (Fig. 3C and D). Although both expression of *PTENP1* and PTEN were concurrently observed in HNSCC cells with stable *PTENP1* expression, the level of PTEN was still insufficient compared with that of the normal epithelial cells, suggesting that *PTENP1* was not sufficient to fully recover the PTEN level in HNSCCs.

### *PTENP1* inhibits the growth and colony formation of HNSCCs

**Figure 4:**
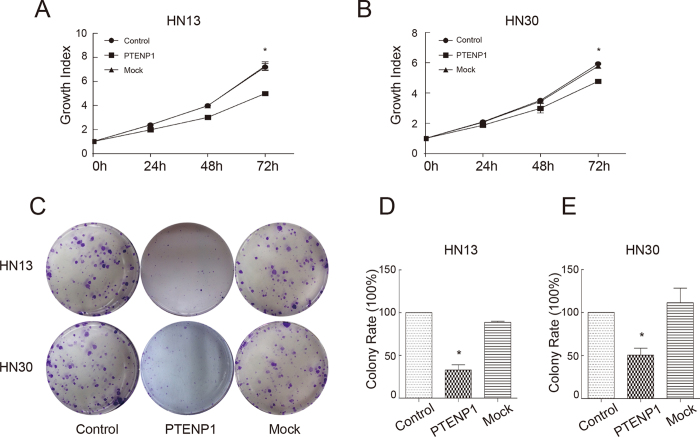
Over-expression of *PTENP1* affects tumour growth and colony formation. (**A**,**B**) The MTT assay for evaluation of the proliferation of HN13 and HN30 cells, respectively. The absorbance was detected at 0 h, 24 h, 48 h and 72 h. The 0 h absorbance of each group was normalized to 1. The relative values were shown as growth index. The data were presented as the means ± SD. *p < 0.05. (**C**) The colony formation assay for evaluation of the proliferation of HN13 and HN30 cells, respectively. (**D**,**E**) Count statistics of the colony formation assay. The colony number of control cells was normalized to 100%. The data were presented as the means ± SD. *p < 0.05.

To decipher the influence of *PTENP1* on the biological functions in HNSCCs, we evaluated the proliferation of tumour cells by the MTT assay. Compared with controls, there was a moderate decrease in cell growth on day 3 when *PTENP1* was expressed in HN13 cells (Fig. 4A). In HN30 cells, *PTENP1* expression led to a mild decrease in cell growth on day 3 (Fig. 4B). In accordance with the findings of the proliferation assay, the colony numbers of *PTENP1*-expressing cells were remarkably decreased (Fig. 4C). Meanwhile, the sizes of the colonies of the *PTENP1*-expressing cells were smaller compared with the large and visible clones in control cultures. The colony formation rate of *PTENP1* cells decreased sharply by approximately 33% and 50%, respectively, in HN13 and HN30 cells (Fig. 4D and E).

### *PTENP1* suppresses the invasion and migration of HNSCCs

**Figure 5:**
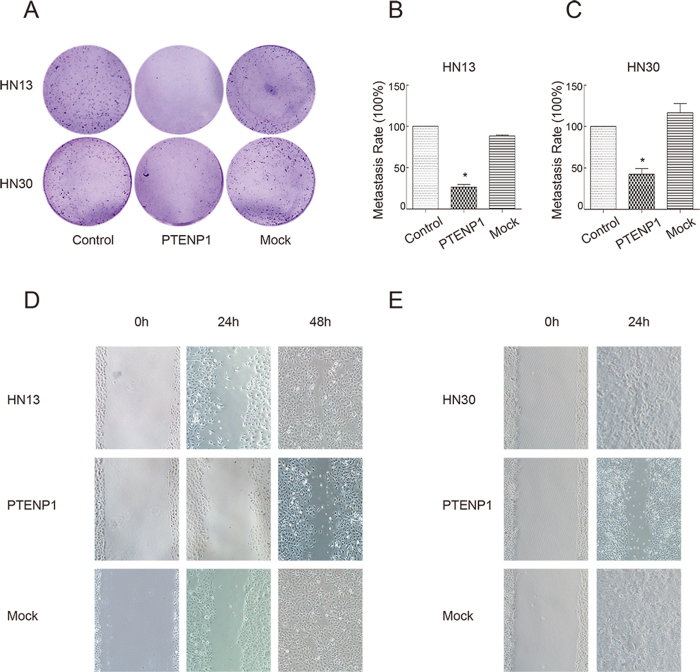
*PTENP1* ectopic expression influences on tumour invasion migration and migration. (**A**) The transwell assay for evaluation of the invasion of HN13 and HN30 cells, respectively. (**B**,**C**) The invasion rate based on the transwell assay. The value of the control was normalized to 100%. The data were presented as the means ± SD. *p < 0.05. (**D**,**E**) The scratch wound assay for evaluation of the migration of HN13 and HN30 cells, respectively.

To assess whether the invasion of HNSCC cells is influenced by *PTENP1*, the transwell assay was performed. The number of migratory cells was significantly reduced in the *PTENP1*-expressing HN13 and HN30 cells (Fig. 5A). The metastasis rate decreased to approximately 26% and 42%, respectively, in the HN13 and HN30 cells (Fig. 5B and C). To evaluate whether the migratory ability of HNSCC cells was influenced by *PTENP1,* the scratch wound assay was also performed. The HN13 control cells at the wound edge migrated into the wound space quickly and merged together at 48 h (Fig. 5D). When *PTENP1* was over-expressed, the cells migrated into only about half of the wound space without merging. The HN30 cells at the wound edge crept across the wound space and merged together at 24 h (Fig. 5E). However, HN30 cells with *PTENP1* migrated slowly without merging.

### *PTENP1* overexpression suppressed tumorigenicity *in vivo*

**Figure 6:**
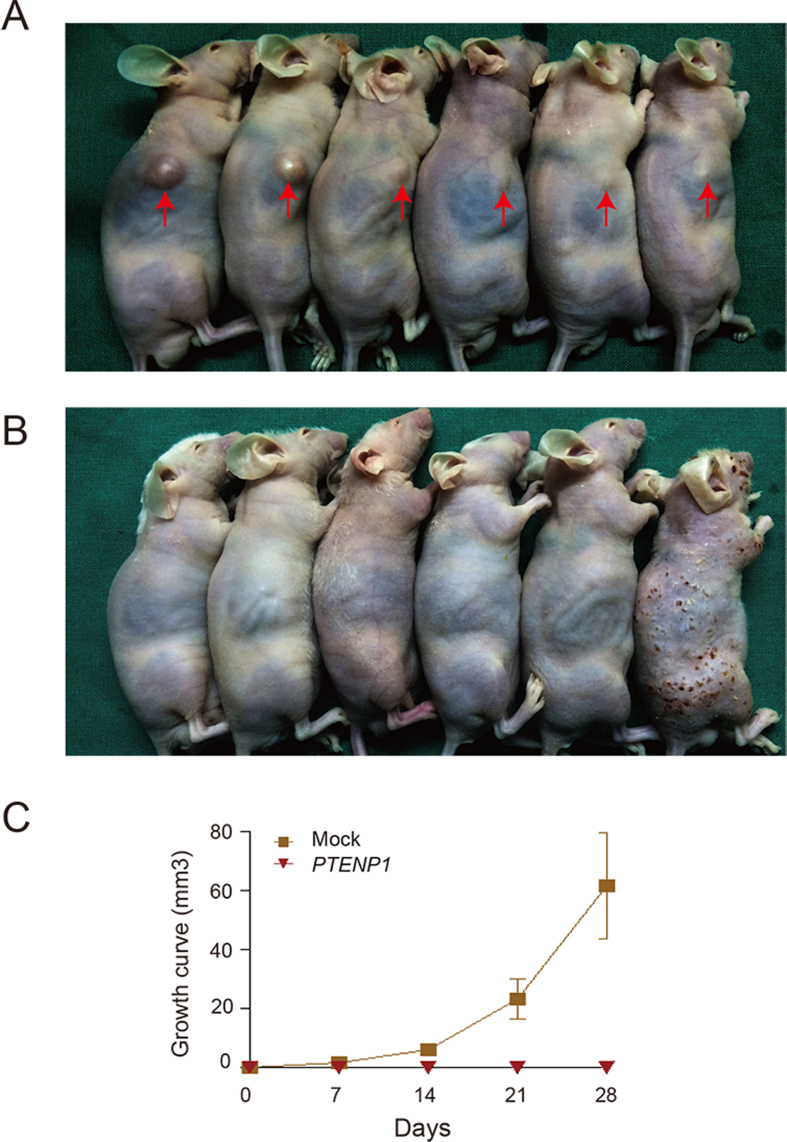
Exogenous expression of *PTENP1* inhibited the growth of xenograft tumour of HN13 cell lines. (**A**,**B**) Lumps of mice carrying Mock cells and *PTENP1* expressing cells in xenograft tumour model, respectively. (**C**) Growth curve based on the xenograft tumour assay. The tumour size was calculated using the formula length *width *width/2. The data were presented as the means ± SD.

To confirm the tumorigenicity of *PTENP1 in vivo*, we established a xenograft model of nude mice using *PTENP1*-enriched HN13 cells and mock control cells. The control cells gradually grew into visible lumps on the flank of mice (Fig. 6A). However animals carrying cells presented expression of the *PTENP1* did not show any visible lump (Fig. 6B). The tumorigenic ability was significantly reduced by *PTENP1* (Fig. 6C). These data further indicated that *PTENP1* could modulate HNSCCs progression *in vivo*.

## Discussion

An increasing number of studies have suggested that dysregulation of lncRNAs in HNSCC. Maternally expressed gene 3 (*MEG3*) was identified as a lncRNA tumour suppressor in a series of cancer types, including tongue squamous cell carcinoma (TSCC) caused by genetic and epigenetic disorders^21^. LncRNA *UCA1* was found to be overexpressed in TSCCs and to promote tumour metastasis^22^. LncRNA *HOTAIR* and *MALAT*-1 were confirmed to be detectable in the saliva of oral squamous cell carcinoma (OSCC) patients, suggesting that lncRNAs may be potential markers for diagnosing cancer^23^.

Recently, lncRNA *PTENP1* expression was found to be decreased in some cancer types, including Hodgkin’s lymphoma, acute myelocytic leukaemia and nasopharyngeal carcinoma^24^,^25^,^26^. *PTENP1* 3′ UTR over-expression resulted in the growth inhibition of cancer cells, suggesting that *PTENP1* plays a tumour suppressive role in prostate cancers. In clear-cell renal cell carcinoma (ccRCC), *PTENP1* could suppress tumour growth and migration^27^. In endometrial cancer, patients with *PTENP1*-positive tumours exhibited a trend towards lower disease recurrence^28^. However, the expression patterns and biological functions of *PTENP1* in HNSCCs have not yet been uncovered. In the present study, we found that ectopic expression of *PTENP1* leads to inhibition of the tumour growth, colony formation, migration and xenograft tumour growth of HNSCC.

In previous studies, the mechanisms underlying the reduced *PTENP1* expression in cancers were mainly divided into two categories: DNA methylation of the *PTENP1* promoter and copy number alterations of *PTENP1* in the genome. *PTENP1* shares 91% sequence identity with a 921 bp region of the PTEN CpG island^16^. The hypermethylation of *PTENP1* has been detected in lymphomas, colorectal cancers, ccRCC and non-small-cell lung cancers (NSCLCs)^29^. In sporadic colon cancer samples, copy number reduction was detectable at the *PTENP1* locus (9p13), independent of nearby locus loss^20^. In addition, partial or complete deletions of the *PTENP1* copy number were also detected in 14.3% of melanoma cell lines and 20.9% of melanoma tissues^19^. In this study, we confirmed that there was copy number reduction in the *PTENP1* locus in 80% of HNSCCs cell lines (one line showed complete deletion, three showed partial deletion and one showed no deletion), suggesting that copy number alterations were the one of main factors affecting the expression of *PTENP1* (Fig. 2).

*PTENP1* was named as a pseudogene of PTEN, which remains the only definite target gene of *PTENP1*. PTEN is a tumour suppressor gene that is dysfunctional in many hereditary and sporadic cancers^30^,^31^. Its expression level was positively correlative with that of *PTENP1*^20^,^27^. Within the high homology region of the PTEN 3′ UTR, *PTENP1* had perfect conserved seed matches for the PTEN-targeting microRNA families, including miR-17, miR-21, miR-214, miR-19 and miR-26 families^20^. *PTENP1* bound PTEN-targeting microRNAs, which protected PTEN mRNA, preventing its degradation by microRNAs. *PTENP1* acted as a decoy for miR-19b and miR-20a in prostate cancer cells and for miR-21 in ccRCC cells^27^,^32^. In the present study, a positive correlation between *PTENP1* expression and the PTEN mRNA level was found in the HNSCC cells as described above. However, the activation induced by *PTENP1* was not potent enough to recover PTEN to a normal level. As the deletion of PTEN was concomitant with that of *PTENP1* in some cancers^19^,^30^, we also examined the genomic PTEN status in HNSCCs. There was only one partial deletion at intron 1 of PTEN in the HN13 cell line. Based on these data, we hypothesize that: 1) *PTENP1* is not a potent activator of PTEN in HNSCCs. As a decoy, its function of protecting PTEN from microRNAs is limited. 2) There are other targets of *PTENP1* in HNSCCs. Of note, even in the absence of PTEN, *PTENP1* still exhibited a tumour suppressive function. Some studies attribute this activity to *PTENP1* acting as a decoy for other miRNA-targeting tumour suppressors^20^. We believe that *PTENP1* is not restricted to serving only a decoy function. As an indispensable partner of epigenetics, the lncRNA can work through multiple mechanisms: establishing gene imprints, orchestrating chromosome conformation, recruiting factors to modulate DNA and histone modification. The mechanisms of action of *PTENP1* in cancers independent of PTEN still warrant further investigation.

## Conclusions

In summary, the expression of *PTENP1* was decreased in both HNSCC cell lines and clinical HNSCC specimens compared with adjacent tissues due to a reduction in the *PTENP1* copy number. Decreased *PTENP1* expression was significantly associated with a worse OS and DFS in HNSCC patients. Overexpression of *PTENP1* significantly inhibited the proliferation, colony formation and migration of HNSCC cells and xenograft tumour growth, suggesting that that *PTENP1* may be a novel therapeutic and prognostic target for HNSCC.

## Methods

### Cell culture

The human HNSCC cell lines (WSU-HN4, HN6, HN13, HN30 and Cal27) were cultured in Dulbecco’s modified Eagle medium (DMEM) (Gibco, Carlsbad, CA, USA supplemented with 10% heat-inactivated FBS (Gibco, Carlsbad, CA, USA) at 37 °C in a humidified 5% CO_2_ atmosphere. Normal oral mucosal epithelial cells were harvested and cultured as described in a previous study^18^.

### Clinical samples

Samples were collected from a cohort of 57 patients who were diagnosed with head and neck squamous cell carcinoma between June 2008 and May 2010. All samples were obtained from the Department of Oral Maxillofacial-Head and Neck Oncology, School of Stomatology, Shanghai Jiao Tong University School of Medicine (Shanghai, China) and quickly frozen in liquid nitrogen upon resection until total RNA was extracted. The clinicopathological characteristics of the study cohort are summarized in Tables 1 and 2. All of the patients provided written informed consent in accordance with the institutional guidelines and this study was approved by Shanghai Ninth People’s Hospital Ethical Committee (No. 2016-172-T121). All methods were performed in accordance with the relevant guidelines and regulations.

RNA extraction, DNA preparation, reverse transcription, RT-PCR, qRT-PCR and genomic qRT-PCR.

Total RNA was extracted from freshly frozen samples and cell lines using the TRIzol Reagent (Invitrogen, Carlsbad, CA, USA), and total RNA was reverse-transcribed with the PrimeScript RT-PCR Kit (TakaraBio, Otsu, Japan). Genomic DNA was extracted using a TIANamp Genomic DNA Kit (Tiangen Biotech, Beijing, China). Cytoplasmic and nuclear RNA was extracted using a Fisher BioReagents SurePrep Nuclear or Cytoplasmic RNA Purification Kit (Thermo Fisher, Waltham, MA, USA). The RT-PCR was performed using Premix Ex Taq reagent (TakaraBio, Otsu, Japan). The qRT-PCR and genomic qRT-PCR were performed using SYBR Select Master Mix (Applied Biosystems, Irvine, CA, USA) and an ABI 7500 real-time PCR system (Applied Biosystems, Irvine, CA, USA). The primer sequences used were as follows: forward 5′-GGATCATTACCTCACACCATACC-3′ and reverse 5′-TCTAAGAAACAACTAAGCCAAAGTC-3′ for *PTENP1*; forward 5′-CTTACAGTTGGGCCCTGTACCATCC-3′ and reverse 5′-TTTGATGCTGCCGGTAAACTCCACT-3′ for PTEN; forward 5′-TGCAGTTTCAGGTACAACACATTGG-3′ and reverse 5′-TCACATACCTTATACACTGGCCTACC-3′ for intron 1 of PTEN; forward 5′-AAGTCAAGAAGTCCAAGAGCATTG-3′ and reverse 5′-TTATGGGCTCAAATATGGGCTAGATG-3′ for intron 3 of PTEN; forward 5′-GGTGGGAAGTATTGCCACTCA-3′ and reverse 5′-GTGAAACCCCAATTTATGTAGCGTAT-3′ for UBE2E1; forward 5′-AGGTCGGTGTGAACGGATTTG-3′ and reverse 5′-TGTAGACCATGTAGTTGAGGTCA-3′ for GAPDH; forward 5′-TATCTGATACGTCCTCTATCCGAGG-3′ for *U2* nuclear RNA. The 2^−ΔΔCT^ equation was used to calculate the relative expression levels.

### Western blot analysis

Cells were rinsed twice with PBS and incubated with RIPA lysis buffer (Millipore, Billerica, MA, USA) on ice for 30 min. Cell extracts were harvested and centrifuged at 13,000 g for 30 min at 4 °C. Then, the lysates were quantified and denatured. Protein samples (10 μg) were separated by sodium dodecyl sulfate–polyacrylamide gel electrophoresis in 10% (wt/vol) polyacrylamide gels and transferred to polyvinylidene fluoride membranes. After blocking, the membrane was incubated with 0.15 μg/ml PTEN antibody (Abcam, Cambridge, MA, USA) overnight at 4 °C or with a β-actin antibody for 1 h (Sigma-Aldrich, St. Louis, MO, USA). The membrane was then incubated with a secondary antibody conjugated to a fluorescent tag (Invitrogen. Carlsbad, CA, USA) for 1 h. The band signals were visualized by the Odyssey Infrared Imagining System (LI-COR) at an 800 channel wavelength.

### Plasmid construction

The 3′ UTR of *PTENP1* was cloned using KOD-Plus-Neo DNA polymerase (TOYOBO, Osaka, Japan) from the genomic DNA of normal oral mucosal epithelial cells. The primers used for cloning were as follows: forward 5′-CGCTACTACCGGAATTCGAGGAGCCGTCAAATCCAGAG-3′ with a BamHI site and reverse 5′-ACTACTACGCGGATCCTCGTCAATGTGTGAGGTTCC-3′ with an EcoRI site. Then, the 3′ UTR of *PTENP1* was inserted into the MCS of the pCDH-CMV-MCS-EF1-Puro lentivirus vector (eukaryotic expression plasmid, PCMV for short) to generate the PCMV-*PTENP1* plasmid (System Biosciences, Palo Alto, CA, USA), and the PCMV with no insertion was called PCMV-Mock used as a control.

### Lentivirus package

When 293 T cells had grown to 70% confluence on a 10 cm disc, 30 μl of Lipofectamine 2000 reagent (Invitrogen, Carlsbad, CA, USA) incubated with Opti-MEM I reduced serum medium (Gibco, Carlsbad, CA, USA) were added, together with 3 μg PCMV-*PTENP1* plasmid or PCMV-Mock plasmid, 3 μg pMD2.D plasmid and 6.0 μg PsPax plasmid, and the cells were incubated for 6 h. The medium was then replaced with 10 ml of fresh medium. The supernatants of cells were collected at 48 h and 72 h. The resulting viruses were filtered through a 0.45-μm cellulose acetate filter, concentrated with Amicon Ultra-15 Centrifugal Filter Units (Millipore, Billerica, MA, USA) and then stored in several aliquots in a −80 °C freezer. HN13 and HN30 cells were seeded at 1.0 × 10^5^ cells per well of six-well plates. One day later, virus (PCMV-*PTENP1* or PCMV-Mock) was added into the medium containing 10 ng/ml polybrene (Sigma-Aldrich, St. Louis, MO, USA). After 48 h, the medium was replaced with fresh medium containing 4 μg/ml puromycin (Invivogen, San Diego, CA, USA), and cells were incubated for at least 2 weeks. Colonies were selected and expanded for further analyses. Stable cell lines with *PTENP1* expression were termed “*PTENP1* cells” and cells with PCMV-Mock were termed “Mock cells”.

### Proliferation assay

The MTT assay was used to detect the proliferative rates of the indicated cell lines. A total of 2,000 *PTENP1* or Mock cells in 100 μl of medium were seeded per well in 96-well plates. At 0 h, 24 h, 48 h and 72 h, 20 μl of 5 mg/ml 3- (4,5-dimethylthiazol-2-yl) -2,5-diphenyltetrazolium bromide (MTT, Sigma-Aldrich, St. Louis, MO, USA) was added to each well. The cells were lysed with 100 μl of dimethylsulfoxide 4 h later with gentle shaking. The optical density was detected with a microplate reader at 490 nm. The absorbance values were normalized to the values of the 0-hour sample. The normalized value was set as the growth index.

### Colony formation assay

Five hundred *PTENP1* or Mock tumour cells were seeded per well in 6-well plates and cultured in complete medium for 10 days. Colonies were then stained with 0.25% crystal violet and colonies of more than 50 cells were counted under a dissecting microscope. The data are shown as the means (±SD) from at least three independent experiments.

### Transwell assay

Ten thousand *PTENP1* or Mock tumour cells were suspended in 400 μl of the appropriate medium supplemented with 2% FBS and were seeded into the upper compartments of a 24-well transwell system with 8-μm pore size polycarbonate filters (Millipore, Billerica, MA, USA). The lower compartments contained 900 μl of medium with 10% FBS. On the second day of incubation, the upper transwells were stained with 0.25% crystal violet. The inner sides of the transwells were scrubbed away and the outer sides were photographed. The crystal violet was washed with 100 μl of 33% acetic acid. The absorbance values of the liquid were detected with a microplate reader at 630 nm.

### Wound-healing assay

*PTENP1* or Mock cells were seeded in 6-well plates with complete medium until they reached full confluence. A wound was then made by using a 200 μl pipette tip to scratch along a line. The cells were rinsed 3 times with PBS to remove suspended cells. Then, the cells were cultured in medium without FBS and the width of the wound was photographed at 0 h, 24 h and 48 h.

### *In vivo* xenograft model

All animal experiments were approved by the Shanghai Jiao Tong University School of Medicine Animal Ethics Committee and this study were conducted following the Shanghai Jiao Tong University School of Medicine animal policy. Stable *PTENP1* or Mock cells were harvested and washed twice with PBS. The cells (5 × 10^6^) were injected subcutaneously into the right flanks of 4-week-old male nude mice in 100 μl of PBS (six mice for each group). The growth of the tumours was observed once a week, and the length and the width of the tumours were measured until four weeks after injection. After euthanasia, the images of mice were stored and the growth curve of tumours was calculated using the formula length*width*width/2.

### Statistical analysis

All of the experiments in our study were performed in triplicate at least. The log-rank test was used to assess the univariate associations between the *PTENP1* transcript level and the Overall survival (OS) and Disease-free survival (DFS). The hazard ratios with corresponding 95% confidence intervals (CIs) and *P* values are reported. For the real-time PCR analysis, the associations between the *PTENP1* mRNA levels and patient characteristics were evaluated using the Kruskal-Wallis test. All tests were two-sided, and *P* values < 0.05 were considered to be statistically significantly. All analyses were conducted using the SPSS software program (standard version 18.0; SPSS Inc., Chicago, Ill).

## Additional Information

**How to cite this article:** Liu, J. *et al*. Decreased expression of pseudogene *PTENP1* promotes malignant behaviours and is associated with the poor survival of patients with HNSCC. *Sci. Rep.* **7**, 41179; doi: 10.1038/srep41179 (2017).

**Publisher's note:** Springer Nature remains neutral with regard to jurisdictional claims in published maps and institutional affiliations.

## Supplementary Material

Supplementary Information

## Figures and Tables

**Table 1:** Association Between PTENP1 RNA Level and Clinical Parameters.

*Characteristics*	*PTENP1* (ΔCT)		
	*No.*	*Mean* ± *SD*	*P*
Age, year	57		
≥60	34	10.69 ± 1.99	0.832
<60	23	10.80 ± 2.12	
Gender	57		
Male	41	10.99 ± 2.05	0.183
Female	16	10.16 ± 1.89	
Smoking history	57		
Smoker	19	11.26 ± 2.19	0.167
Non-smoker	39	10.47 ± 1.91	
Alcohol history	57		
Drinker	16	11.63 ± 2.08	0.034
Non-drinker	41	10.38 ± 1.91	
Disease site	57		
Oral cavity	49	10.67 ± 2.09	0.548
Oropharynx	8	11.14 ± 1.64	
Tumor status	57		
Primary	50	10.70 ± 2.11	0.718
Recurrence	7	11.00 ± 1.28	
Tumor size	52		
≥2 cm	48	10.93 ± 1.92	0.123
<2 cm	4	9.35 ± 2.17	
TNM Stage	53		
I–II	15	10.22 ± 2.02	0.177
III–IV	38	11.02 ± 1.89	
Tumor grade	54		
I–II	47	10.77 ± 2.05	0.591
III	7	11.20 ± 1.29	
Lymph node metastasis	57		
Negative	28	10.57 ± 1.96	0.545
Positive	29	10.89 ± 2.10	

Abbreviations: SD, standard deviation; TNM stage, tumor-node-metastasis stage; ΔCT indicates the difference in the cycle number at which a sample’s fluorescent signal passes a given threshold above baseline (Ct) derived from a specific gene compared with that of GAPDH.

**Table 2:** Univariate Cox Proportional Hazards Regression Models for Estimating Overall Survival and Disease-Free Survival.

*Characteristics*	*Overall Survival*			*Disease-Free Survival*		
	HR	95%Cl	P	HR	95%Cl	P
Age (≥60 vs <60)	0.623	0.187 to 2.076	0.441	0.895	0.289 to 2.772	0.847
Gender (Male vs Female)	0.673	0.184 to 2.460	0.549	0.675	0.186 to 2.443	0.549
Tumor status (Primary vs Recurrence)	0.883	0.189 to 4.126	0.874	1.050	0.208 to 5.311	0.953
Disease site (Oral cavity vs Oropharynx)	0.781	0.157 to 3.880	0.762	0.527	0.106 to 2.634	0.435
Smoking history (Smoker vs Nonsmoker)	1.224	0.247 to 6.070	0.805	1.063	0.221 to 5.108	0.940
Alcohol history (Drinker vs Nondrinker)	1.094	0.212 to 5.643	0.914	0.721	0.147 to 3.536	0.687
Tumor size (≥2 cm vs <2 cm)	0.379	0.034 to 4.254	0.432	0.533	0.047 to 6.054	0.612
TNM stage (I-II vs III-IV)	0.469	0.090 to 2.441	0.369	0.334	0.067 to 1.652	0.179
PTENP1 expression (High vs Low)	0.170	0.049 to 0.590	0.005	0.195	0.057 to 0.664	0.009
Tumor grade (I–II vs III)	1.432	0.315 to 6.520	0.642	1.577	0.354 to 7.028	0.550
Lymph node metastasis (Positive vs Negative)	1.320	0.318 to 5.484	0.702	2.153	0.603 to 7.688	0.238

Abbreviations: CI, confidence interval; HR, hazard ratio; TNM, tumor-lymph node-metastasis classification.
