# Supplementary material for: Decreased expression of pseudogene PTENP1 promotes malignant behaviours and is associated with the poor survival of patients with HNSCC
Source: Sci Rep. 2017 Jan 23;7:41179. doi: 10.1038/srep41179 (PMC5255549; doi:10.1038/srep41179)
Supplement: Supplementary Information [file srep41179-s1.doc]

Decreased expression of pseudogene PTENP1 promotes malignant behaviours and is associated with the poor survival of patients with HNSCC

Jiannan Liu ^1,2^† First author

**1. Department of Oral Maxillofacial-Head and Neck Oncology**, Ninth People’s Hospital, Shanghai Jiao Tong University School of Medicine, Shanghai 200011, P.R. China

2. Shanghai Research Institute of Stomatology and Shanghai Key Laboratory of Stomatology, Shanghai 200011, China

E-mail:laurence_ljn@163.com

Yue Xing ^3^† Co-first author

**3.Department of Ophthalmology**, Ninth People’s Hospital, Shanghai Jiao Tong University School of Medicine, Shanghai, P.R. China

E-mail: fishashorejessica@163.com

Liqun Xu ^1,2^† Co-first author

**1. Department of Oral Maxillofacial-Head and Neck Oncology**, Ninth People’s Hospital, Shanghai Jiao Tong University School of Medicine, Shanghai 200011, P.R. China

2. Shanghai Research Institute of Stomatology and Shanghai Key Laboratory of Stomatology, Shanghai 200011, China

E-mail: maxilla@sina.com

Wantao Chen ^1,2^ Co-author

**1. Department of Oral Maxillofacial-Head and Neck Oncology**, Ninth People’s Hospital, Shanghai Jiao Tong University School of Medicine, Shanghai 200011, P.R. China

2. Shanghai Research Institute of Stomatology and Shanghai Key Laboratory of Stomatology, Shanghai 200011, China

E-mail: chenwantao196323@sju.edu.cn

Wei Cao ^1,2^ * Corresponding author

**1. Department of Oral Maxillofacial-Head and Neck Oncology**, Ninth People’s Hospital, Shanghai Jiao Tong University School of Medicine, Shanghai 200011, P.R. China

2. Shanghai Research Institute of Stomatology and Shanghai Key Laboratory of Stomatology, Shanghai 200011, China

E-mail: caowei561521@hotmail.com

The first three authors were equally contribute to this work.

Chenping Zhang ^1,2^ * Corresponding author

**1. Department of Oral Maxillofacial-Head and Neck Oncology**, Ninth People’s Hospital, Shanghai Jiao Tong University School of Medicine, Shanghai 200011, P.R. China

2. Shanghai Research Institute of Stomatology and Shanghai Key Laboratory of Stomatology, Shanghai 200011, China

E-mail: zhang.chenping@hotmail.com


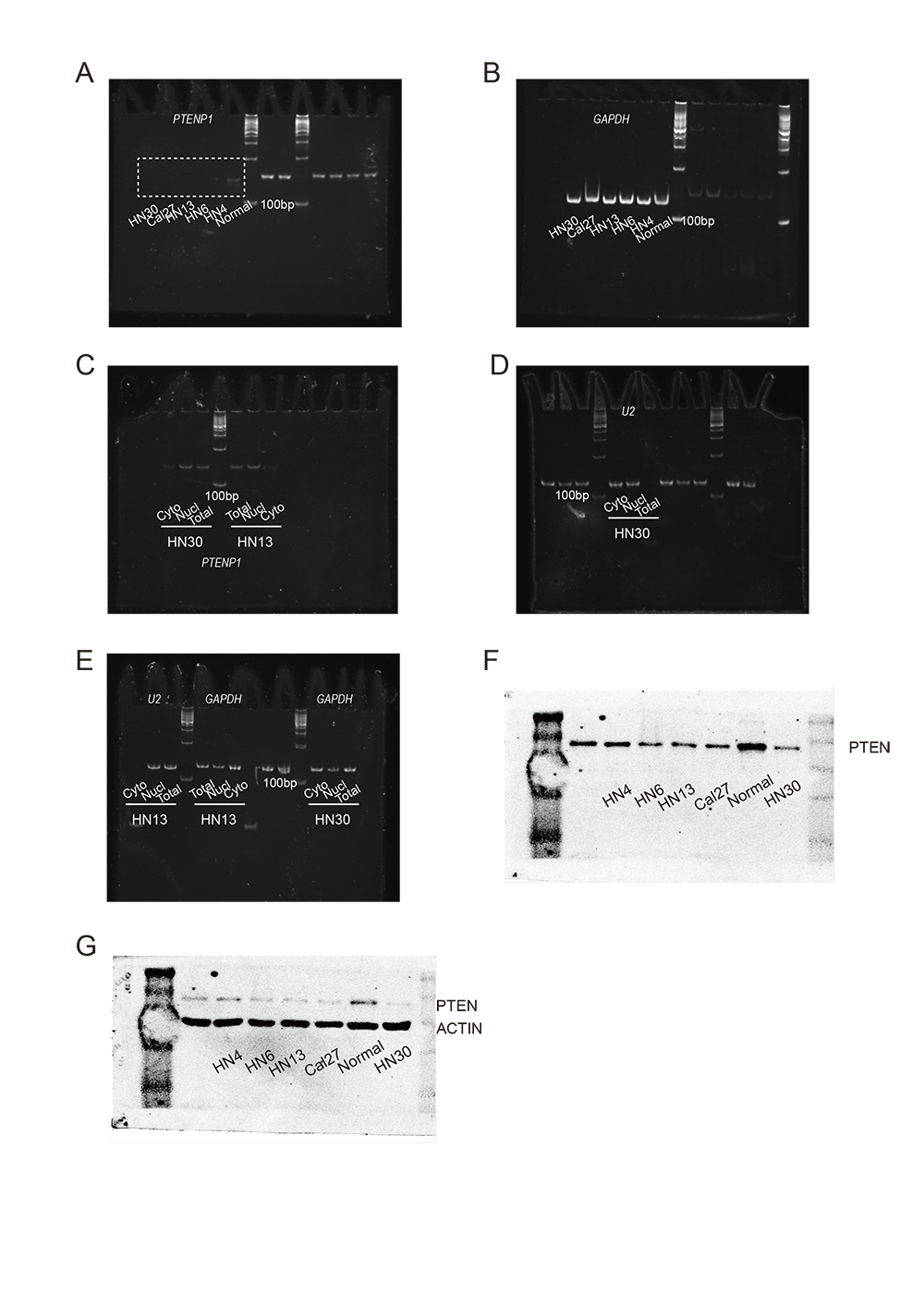


Supplementary Figure legends

A-B: Full-length gels for Figure 1A.

C-E: Full-length gels for Figure 1D.

F-G: Full-length blots for Figure 2B.
